# Navigation and Load Adaptability of a Flatworm-Inspired Soft Robot Actuated by Staggered Magnetization Structure

**DOI:** 10.3390/biomimetics11010041

**Published:** 2026-01-06

**Authors:** Zixu Wang, Miaozhang Shen, Chunying Li, Pengcheng Li, Anran Zheng, Shuxiang Guo

**Affiliations:** 1 Department of Electronic and Electrical Engineering, Southern University of Science and Technology, Shenzhen 518055, China; wangzx3@sustech.edu.cn (Z.W.); shenmz2024@mail.sustech.edu.cn (M.S.); lipc@sustech.edu.cn (P.L.); 2 Advanced Institute for Ocean Research, Southern University of Science and Technology, Shenzhen 518055, China; licy@sustech.edu.cn; 3 CAS Key Lab of Bio-Medical Diagnostics, Suzhou Institute of Biomedical Engineering and Technology, Chinese Academy of Sciences, No. 88, Keling Road, Suzhou 215163, China; zhengar@sibet.ac.cn; 4 Aerospace Center Hospital, School of Life Science and the Key Laboratory of Convergence Medical Engineering System and Healthcare Technology, Ministry of Industry and Information Technology, Beijing Institute of Technology, Beijing 100081, China

**Keywords:** magnetically actuated minirobot, electromagnetic field, traveling wave locomotion, flatworm-inspired soft robot

## Abstract

This study presents a magnetically actuated soft robot inspired by the peristaltic locomotion of flatworms, designed to replicate the biological locomotion of worms to achieve robust maneuverability. Fabricated entirely from photocurable soft resin, the robot features a flexible elastomeric body and two webbed fins with embedded soft magnets. By applying a vertically oscillating magnetic field, the robot achieves forward crawling through the coordinated bending and lifting of fins, converting oscillating magnetic fields into continuous undulatory motion that mimics the gait of flatworms. The experimental results demonstrate that the system maintains consistent bidirectional velocities in the range of 4–7 mm/s on flat surfaces. Beyond linear locomotion, the robot demonstrates effective terrain adaptability, navigating complex topographies, including curved obstacles up to 16 times its body thickness, by autonomously adopting a high-lifting kinematic strategy to overcome gravitational resistance. Furthermore, load-carrying tests reveal that the robot can transport a 6 g payload without velocity degradation. These findings underscore the robot’s efficacy in overcoming mobility constraints, highlighting promising applications in fields requiring non-invasive intervention, such as biomedical capsule endoscopy and industrial pipeline inspection.

## 1. Introduction

Recent advances in microrobotic technology have facilitated the development of minimally invasive devices and high safety is being progressively developed and applied within human anatomical cavities. Concurrently, the intrinsic flexibility and functional advantages observed in natural organisms, such as octopuses, jellyfish, and caterpillars, have inspired the integration of soft materials into the design of bioinspired robots. These soft robots exhibit exceptional adaptability when performing complex tasks, especially in confined or cluttered environments where traditional rigid robots face significant limitations [1,2,3,4]. Their compliant bodies enable safe and efficient operation within narrow spaces, making them highly promising for medical applications, such as navigating through delicate anatomical regions inside the human body [5,6,7,8]. Moreover, the wireless controllability of such systems enhances patient comfort and shortens recovery times compared to conventional invasive procedures [9]. As a result, soft robots are being widely investigated for biomedical uses, including targeted drug delivery and minimally invasive surgery [10,11,12].

To achieve locomotion in these systems, various actuation stimuli have been explored, ranging from pneumatic pressurization and dielectric elastomer actuation to light-driven mechanisms [13,14,15,16,17]. Li et al. introduced a miniature coiled artificial muscle actuator that utilizes radio frequency-magnetic heating for wireless control, which significantly surpasses the capabilities of traditional magnetic soft actuators. This study enables the development of versatile wireless medical devices, such as suturing tools, scissors, and drillers [18]. Among these modalities, magnetic actuation has emerged as a particularly advantageous solution for biomedical scenarios. Unlike pneumatic tethering or thermal actuation that may damage tissues, magnetic fields offer safe, instantaneous, and untethered control with deep tissue penetrability [19,20]. Consequently, recent research has rapidly advanced the field of magnetically actuated soft robots. For instance, the technique of programming ferromagnetic domains into soft materials, pioneered by Kim et al., has enabled robots to execute complex multimodal shape transformations [21]. Wu et al. introduced magnetic soft actuators leveraging liquid crystalline elastomers with covalent adaptable networks, which enable reprogrammable contraction-derived motions such as bidirectional shrinkage and dynamic 3D patterns through magnetothermal responsiveness. Their study offers advantages including stepwise controllability, multiresponsiveness, self-healing, and remolding ability for versatile applications in confined spaces [22]. Utilizing such principles, researchers have developed various bioinspired prototypes, such as millipedes capable of walking on wet surfaces [23], jellyfish robots that swim through fluidic pulses [24,25], and origami-based sheets that roll and flip [20].

Despite these advancements, effectively replicating the stable and efficient undulatory locomotion seen in nature remains a significant challenge. Many existing magnetic soft robots rely on simple bending or folding kinematics, which often result in discrete, jerky movements or limited load-carrying capacities. While some multi-segmented designs have achieved linear crawling, they typically require complex magnetization profiles or intricate magnetic coil arrays for phase control [26,27]. There is a critical need for a streamlined structural design that can transform a simple uniform driving field into a continuous traveling wave, thereby ensuring both robust obstacle navigability and structural stability for payload transport. While numerous magnetic soft crawlers have been developed, a distinct performance gap remains between robots driven by simple uniform fields and those driven by complex gradients. Monolithic designs under uniform fields typically exhibit standing wave deformation or rolling gaits [12,18,26]. While efficient on flat ground, these kinematic modes often lack the vertical clearance to step over high obstacles and the stability required to balance payloads without tipping. Thus, there is a critical need for a streamlined structural design that can transform a simple uniform driving field into a high-lifting, continuous traveling wave, thereby bridging the simplicity of uniform actuation with the maneuverability of complex gaits.

In magnetic soft robotics, a persistent engineering challenge lies in generating continuous, coordinated traveling waves without relying on complex, cumbersome magnetic gradient coil arrays. Simple uniform fields typically yield only synchronized bending, which significantly limits terrain adaptability. To address this, we present a novel magnetically actuated soft robot inspired by the peristaltic locomotion of flatworms, with the specific engineering goal of decoupling control complexity from actuation hardware.

By translating the flatworm’s muscular architecture into a synergistic structural design, we effectively pre-program the required gait dynamics into the soft body. The core novelty of this work is the implementation of a staggered magnetization scheme within a segmented three-part architecture connected by flexible elastomeric hinges. Specifically, by embedding magnets with alternating polarities into the anterior and posterior segments while leaving the central bridge passive, we establish an efficient “Active-Passive-Active” driving mechanism. This configuration allows the central soft hinge to dynamically arch and store elastic energy, effectively serving as a structural bridge to support external loads while facilitating phase-lagged wave propagation. This strategy advances the state of the art by transforming a simple, globally uniform oscillating magnetic field into complex, high-lifting maneuvers and coordinated propulsion.

## 2. Principle of Operation and Mathematical Model

**Figure 1:**
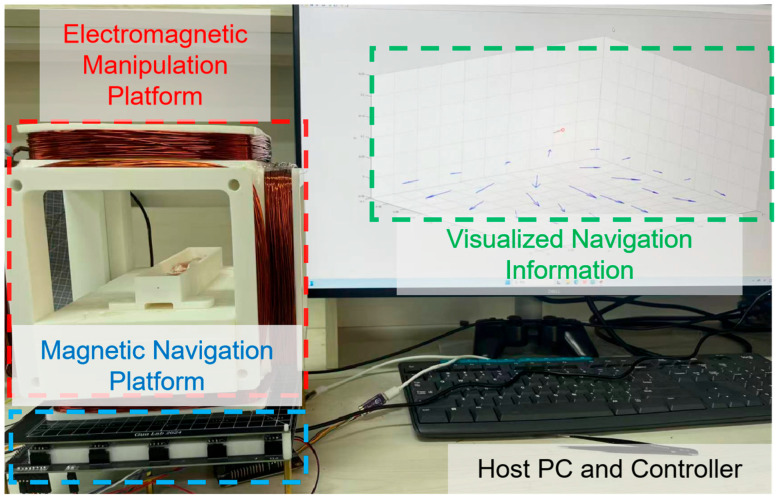
Experimental setup for electromagnetic manipulation. The platform consists of a three-axis Helmholtz-like coil system for generating programmable magnetic fields, a fluid-filled tank serving as the motion workspace.

The experimental system consists of three main components: a magnetic field actuation platform, a navigation platform and an observation system, as shown in Figure 1 and Table 1. The actuation platform is composed of a three-axis Helmholtz-like electromagnetic coil platform, capable of generating a precisely programmable and uniform magnetic field within its central workspace. This system provides the time-harmonic oscillating magnetic field required for robot actuation. The motion environment is a transparent acrylic tube with an inner diameter of 20 mm, filled with water. The observation system utilizes a high-frame-rate CCD to record the robot’s motion from a lateral view.

### 2.1. Structural Design and Actuation Principle

**Figure 2:**
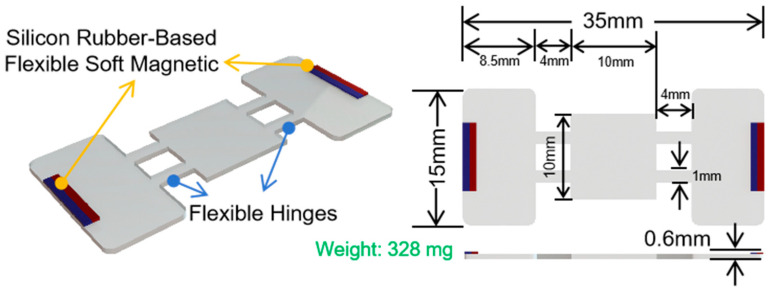
Schematic diagram of the biomimetic soft flatworm robot. The design illustrates the segmented structure consisting of a silicone rubber-based soft body and flexible hinges. Soft magnets are embedded within the top and tail with staggered magnetization directions.

**Figure 3:**
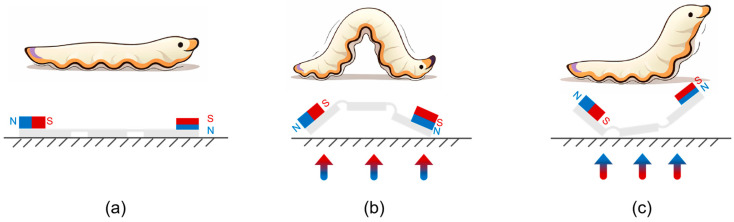
Bio-inspiration and actuation principle. (**a**) The natural creeping locomotion of a flatworm/caterpillar. (**b**) Illustration of the flatworm gait characterized by periodic arching and extension. (**c**) The magnetic actuation mechanism: the interaction between the staggered magnetic poles and the external oscillating field generates alternating torques, inducing a traveling wave motion.

The robot body consists of three webbed fins, with the 1st and 3rd fins embedded with a soft permanent magnet. The first soft magnet is oriented with the north poles facing upward, while the second soft magnet is reversed 90°. The two magnets on the top and tail share different magnetization directions; the schematic diagram of the biomimetic soft flatworm robot is shown in Figure 2. And the actuation principle is shown in Figure 3.

The robot’s motion is driven by an external, uniform oscillating magnetic field, B(t), applied perpendicularly to the robot’s main axis:

(1) $$B(t)=B_{0}sin(\omega t)$$

where $B_{0}$ is the amplitude and ω is the angular frequency. Under this field, each permanent magnet, modeled as a magnetic dipole, experiences a periodic magnetic torque $\tau_{mag}$. This torque is related to the magnetic dipole moment m and the external magnetic field B(t) by:

Under this field, each magnet experiences a periodic magnetic torque $\tau_{mag}$ [12,28]:

(2) $$\tau_{mag}=m\times \;B(t)$$

where $m_{i}$ is the magnetic dipole moment of the magnet. Due to the staggered nature of the magnet arrangement, adjacent fin segments experience torques in opposite directions, inducing an out-of-phase bending response.

For a uniformly magnetized magnet, the magnitude of the magnetic dipole moment m can be approximately estimated as:

(3) $$m\approx \frac{B_{r}}{\mu_{0}}V$$

where $B_{r}$ is the remanent flux density, $\mu_{0}$ is the magnetic permeability of vacuum, and V is the volume of the magnet. Due to the staggered magnet arrangement, adjacent fin segments experience torques in opposite directions, inducing an out-of-phase bending response. This design forms a classic “active-passive-active” architecture, where the head and tail ends are directly actuated. Due to the elasticity of the flexible hinges and fluid–structure interaction, the motion of the central segment exhibits a phase lag relative to the actuated segments. This intrinsically generated phase difference enables the discrete oscillations of the three fins to organically combine and form a macroscopic, continuous, backward-propagating mechanical wave, known as a traveling wave.

### 2.2. Dynamic Modeling

To mathematically describe the robot’s motion, we establish models at two different scales. First, the bending behavior of a single flexible segment can be approximated using a lumped-parameter model. This model simplifies the dynamic response of a fin segment into a second-order system:

(4) $$I_{i}{\ddot{\theta }}_{i}+c{\dot{\theta }}_{i}+{k\theta }_{i}=mB_{0}\sin\left(\omega t\right)$$

where $I_{i}$ is the moment of inertia of the segment, c is the viscoelastic damping coefficient, k is the equivalent torsional stiffness of the flexible hinge, and θ is the bending angle. This model is useful for understanding the fundamental response of an individual actuation unit.

However, a distributed-parameter model is required to more accurately describe the formation and propagation of the traveling wave along the entire body. Therefore, we model the robot’s flexible body as a continuous flexible beam. Its transverse deformation, y(x,t), under the influence of the magnetic field and fluid can be described by a modified Euler-Bernoulli beam equation:

(5) $$EI=\frac{\partial^{4}y(x,t)}{\partial x^{4}}+c\frac{\partial y(x,t)}{\partial t}+\rho A\frac{\partial^{2}y(x,t)}{\partial t^{2}}=m(x)B_{0}\sin\left(\omega t\right)$$

where EI is the beam’s flexural rigidity, ρA is the mass per unit length, c is the viscoelastic damping coefficient, and m(x) is the magnetic moment density distributed along the beam’s length. The solution to this equation, y(x,t), describes the complete spatiotemporal form of the traveling wave [26]. Its solution is typically represented by a superposition of vibrational modes:

(6) $$y\;(x,t)=\;\sum_{n}^{i}Y_{n}(x)\sin\left(\omega_{n}t\;+\;\varphi_{n}\right)$$

where $Y_{n}(x)$ are the mode shapes and $\varphi_{n}$ is the phase difference between different modes. It is the spatial manifestation of this phase difference that generates the traveling wave which propels the fluid.

### 2.3. Propulsion Mechanism

The physical basis for how the traveling wave predicted by the above models generates net thrust is anisotropic fluid drag. In low-Reynolds-number regimes, the normal fluid drag $F_{⊥}$, experienced when a body segment moves perpendicular to its local axis, is significantly greater than the tangential drag F, experienced when it moves parallel to it. During the robot’s motion, the backward-propagating wave effectively pushes the fluid backward via its normal velocity component. According to Newton’s third law, the fluid exerts an equal and opposite reaction force on the robot, and the forward component of this force constitutes the primary propulsive thrust. The net effect of this process can be approximated by a simplified velocity equation:

(7) $$V\approx \frac{\omega L}{\pi }\;(△C_{f}-△C_{b})$$

where L is the wavelength and $△C_{f}$ and $△C_{b}$ represent the difference in friction coefficients.

Because analytically solving the fluid–structure interaction terms in the beam Equation (5) is extremely difficult; we employ the Finite Element Method for numerical simulation. The simulation model is established based on key assumptions: the external magnetic field is uniform; the soft material is hyperelastic; the fluid is an incompressible Newtonian fluid; and inter-magnet interactions are neglected. By solving the fully coupled fluid–structure interaction model in commercial software, we can accurately reproduce the robot’s dynamic response under various actuation parameters, providing theoretical guidance for its optimal design and control strategies.

### 2.4. FEM Simulation

To validate the traveling wave propulsion mechanism predicted by our theoretical model and to quantify its hydrodynamic performance, a series of transient fluid–structure interaction simulations were conducted using the commercial finite element software ANSYS Fluent 2023. We employed an efficient one-way coupling strategy: the kinematics predicted by the theoretical model were used as a prescribed boundary motion, and the interaction between the undulating robot body and the fluid was studied using the Dynamic Mesh technique. Since the magnetic driving force is significantly larger than the fluid drag in this low-Reynolds-number regime, we assume the robot’s deformation is primarily governed by magnetic torque. Thus, a one-way coupling approach is computationally efficient and sufficient to capture the hydrodynamic trends. The motion pattern of the robot’s fins is directly derived from the traveling wave solution Equation (6) of our continuous beam model Equation (5) and is prescribed by a classic traveling wave function for the transverse displacement y (x,t) of any point x on its surface at time t:

(8) y (x,t)= A sin(kx−ωt)

The parameters of this equation are tightly coupled with the physical system: the amplitude A corresponds to the magnitude of the external magnetic field $B_{0}$, the angular frequency ω is the driving frequency, and the wavenumber k is co-determined by the robot’s physical properties and the driving frequency. In this manner, our simulation input forms a direct mapping with the theoretical model.

**Figure 4:**
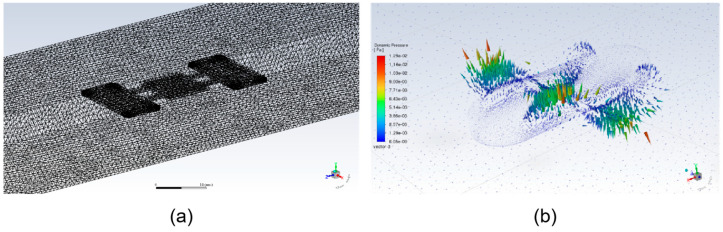
Fluent simulation. (**a**) The computational mesh grid generated for the fluid–structure interaction analysis using ANSYS Fluent. (**b**) Visualization of velocity streamlines showing the fluid flow disturbance induced by the robot’s undulatory fin motion.

In the simulation setup, the robot model was immersed within a rectangular computational domain, where the unstructured tetrahedral mesh was locally refined near the robot’s surface to accurately resolve flow details within the boundary layer. The boundary conditions were defined by setting the robot and channel surfaces as no-slip walls, while the domain extremities were designated as pressure outlets to simulate an open environment. The robot’s undulatory locomotion was implemented via the UDF controlling the dynamic mesh; this function calculates nodal positions for each time step by modulating the static bending deformation profile derived from prior static analysis according to Equation (8), thereby driving the traveling wave kinematics. To ensure mesh quality and convergence during large deformations, a combination of smoothing and remeshing techniques was employed. However, it is acknowledged that this kinematic-driven approach represents an idealized model prediction. Unlike the physical scenario where deformation arises from the real-time dynamic equilibrium of magnetic torque, material viscoelasticity, and fluid drag, this simulation decouples the magnetic-structural interaction to reduce computational complexity. Consequently, this simplification accounts for the inevitable discrepancies observed between the numerical predictions and experimental measurements shown in Figure 4.

## 3. Experiment and Results

### 3.1. Bending Deformation Evaluation

**Figure 5:**
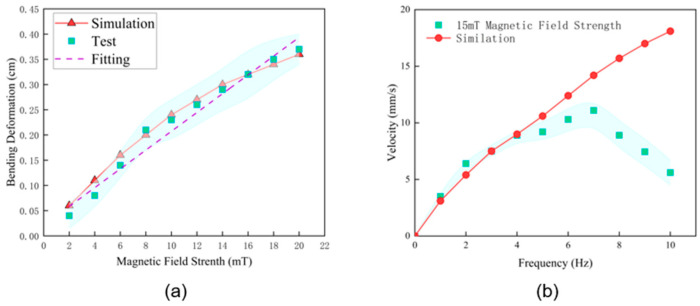
Quantitative characterization of robot performance. (**a**) Comparison between experimental measurements and simulation results of static bending deformation under varying magnetic field strengths (2–20 mT). (**b**) Forward velocity versus actuation frequency under a constant 15 mT field, exhibiting a peak velocity at the resonant frequency of approximately 7 Hz.

To precisely quantify the fundamental actuation performance of the soft robot, its bending deformation under a static magnetic field from 0 to 20 mT was tested, simulated, and analyzed. Figure 5a displays the results from the experimental measurements, the finite element simulation, and a linear regression on the experimental data. It can be observed from Figure 5a that, across the entire tested range, the robot’s bending deformation increases monotonically and approximately linearly with the magnetic field strength. This measurable static bending amplitude serves as the kinematic basis for the robot’s ‘high-lifting’ gait, enabling the clearance height demonstrated in later obstacle experiments.

To further unveil the underlying characteristics, a linear regression analysis was performed on the experimental data. The analysis yielded a very high coefficient of determination, $R^{2}$ = 0.97, which indicates a strong linear correlation between the bending deformation and the magnetic field strength. The fitted line’s slope, b, was determined to be 0.0186 (cm/mT). Physically, this slope quantifies the static actuation sensitivity of the robot, meaning that for every 1 mT increase in field strength, the resulting bending deformation is approximately 0.0186 cm. This highly linear response is a key advantage of the soft robot, as it implies that we can achieve precise and predictable control over the robot’s posture by simply modulating the magnetic field strength linearly. However, this stable linear response is not unlimited. As observed in our dynamic performance tests, when the magnetic field strength approaches or exceeds 20 mT, the robot is prone to an unstable flipping motion due to excessive magnetic torque. Therefore, the 0–20 mT range characterized in this static test not only defines a linear response region but can also be defined as the robot’s stable control operational range.

### 3.2. Velocity and Driving Frequency Evaluation

Actuation frequency was identified as a critical determinant of locomotion performance. To quantify this, we measured the robot’s forward velocity across a range of 0 to 10 Hz under a constant 15 mT magnetic field (Figure 5b). Experimentally, the results exhibit a distinct non-monotonic trend, which contrasts with the linearly increasing prediction from the numerical simulation. In the low-frequency regime (0–7 Hz), velocity rises steadily with frequency, peaking at a maximum of 11.1 mm/s at 7 Hz, thus marking 7 Hz as the optimal operating point. The systematic overestimation in the simulation results likely stems from the model’s idealized linear elastic assumptions, which do not fully account for the complex fluid drag and internal structural damping present in the physical prototype.

The observed velocity profile is governed by the dynamic interplay between structural resonance and material viscoelasticity. Below the 7 Hz threshold, the system operates in a linear kinematic region where a higher stroke rate directly translates to more propulsive cycles per second. The peak performance at 7 Hz represents a resonant-like coupling state, where the external magnetic drive aligns efficiently with the soft body’s natural frequency, maximizing deformation amplitude. However, performance degrades significantly beyond this optimum due to two factors. First, viscoelastic damping intensifies at high frequencies, causing the soft resin to dissipate input energy via internal friction rather than converting it into deformation. Second, kinematic lag occurs when the rapid magnetic switching outpaces the mechanical relaxation time of the passive central segment. This disrupts the phase synchronization required for the traveling wave, degrading effective propulsion into inefficient local vibration.

### 3.3. Velocity and Magnetic Field Strength Evaluation

**Figure 6:**
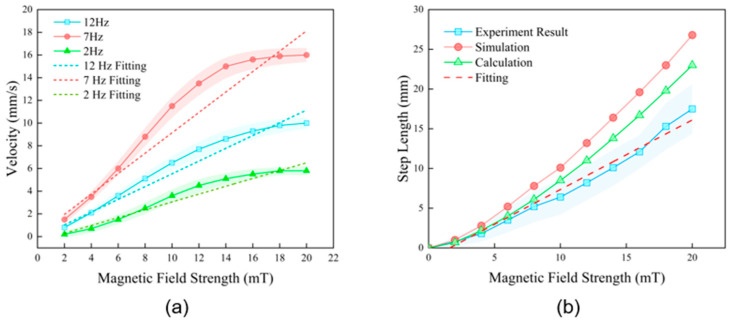
Influence of magnetic field intensity on locomotion performance. (**a**) Forward velocity plotted against magnetic field strength at different driving frequencies (2 Hz, 7 Hz, and 12 Hz), showing a linear response region. (**b**) The step length per cycle as a function of field strength; simulation predictions align well with experimental data.

To systematically investigate the combined effects of actuation parameters on the robot’s locomotion performance, we measured its forward velocity as a function of the magnetic field strength at different driving frequencies (2 Hz, 7 Hz, and 12 Hz). The results are shown in Figure 6a.

The experimental results clearly reveal two core principles. First, the driving frequency is a critical factor that determines the robot’s locomotion efficiency. At any given magnetic field strength, the velocity at 7 Hz is significantly higher than at 12 Hz and 2 Hz. For instance, at a field strength of 20 mT, the velocity reached 16.0 mm/s at 7 Hz, compared to only 10.0 mm/s at 12 Hz and 5.8 mm/s at 2 Hz. This indicates that the robot’s optimal operating frequency is in the vicinity of 7 Hz, where the propulsion efficiency from its traveling wave gait is maximized.

Second, for any given frequency, the robot’s velocity increases monotonically with the magnetic field strength, but this relationship is not infinitely linear. This saturation effect is particularly evident in the optimal 7 Hz case: as the field strength increases from 2 to 20 mT, the forward velocity increases significantly, as a stronger field induces a larger traveling wave amplitude. However, when the field strength exceeds 20 mT, the velocity approaches saturation. This is primarily because the flexible hinges reach their maximum physical bending angle; further increases in the magnetic torque do not lead to a significant increase in the overall deformation amplitude. Furthermore, continuing to increase the magnetic field intensity may cause the segments to flip, resulting in the magnets at the head or tail being reversely attracted to the middle segment, thereby rendering the soft robot immobile.

To quantify the robot’s response sensitivity to magnetic control, we performed a linear regression analysis on the primary linear region of each dataset. The results show high coefficients of determination (all R^2^ > 0.96), indicating a strong linear relationship between velocity and field strength within their non-saturated ranges. The most crucial metric from this analysis is the slope of the fitted line, which represents the velocity sensitivity to the magnetic field. The analysis reveals that the slope is steepest at 7 Hz, reaching 0.899 (mm/s)/mT, which is substantially higher than the 0.562 (mm/s)/mT at 12 Hz and 0.345 (mm/s)/mT at 2 Hz. In summary, 7 Hz is not only the optimal frequency for achieving the highest absolute velocity but also the frequency that provides the greatest sensitivity to magnetic control.

### 3.4. Step Length Evaluation

The result and data in Figure 6b are a comparative relationship between the experimental measurements, theoretical calculations, and numerical simulation predictions. An evident core trend is that, across all three methods, the robot’s step length exhibits a strong positive correlation with the magnetic field strength. This confirms that the robot’s displacement capability can be effectively controlled by tuning the external magnetic field. Concurrently, the overall trends of the three curves show good agreement, mutually validating the reliability of our theoretical framework, numerical simulations, and physical experiments.

Upon quantitative comparison, a systematic difference is observed: at the same magnetic field strength, the simulation predicts the largest step length, followed by the theoretical calculation, while the experimental result is the most conservative. This discrepancy is expected and can be reasonable by the intrinsic characteristics of each method. Numerical simulations are often conducted in idealized physical environments and may not fully account for all forms of energy dissipation, such as minute friction with the pipe bottom or internal damping from material viscoelasticity; thus, their results often represent an upper-bound prediction of performance. The theoretical calculation, based on a simplified physical model, captures the core physical principles but may simplify complex fluid–structure interactions, leading to results that lie between the ideal simulation and the real-world experiment. The experimental result, serving as the ground truth, incorporates all non-ideal factors present in the real world, hence its more conservative step length values.

To further quantify the actual response characteristics of the robot with precision, a linear regression analysis was performed on the experimental data points. The analysis yielded a very high coefficient of determination, $R^{2}$ = 0.977, which indicates a highly linear relationship between the step length and the magnetic field strength within the tested range of 0–20 mT. The slope b of the fitted line was determined to be 0.875 (mm/mT). This slope value intuitively represents the robot’s locomotion sensitivity, meaning that for every 1 mT increase in field strength, its single-cycle forward step length increases by approximately 0.875 mm. This linear characteristic is crucial, as it implies that we can achieve precise and predictable control over the robot’s displacement by simply modulating the magnetic field strength linearly.

### 3.5. Reciprocating Motion Experiment

**Figure 7:**
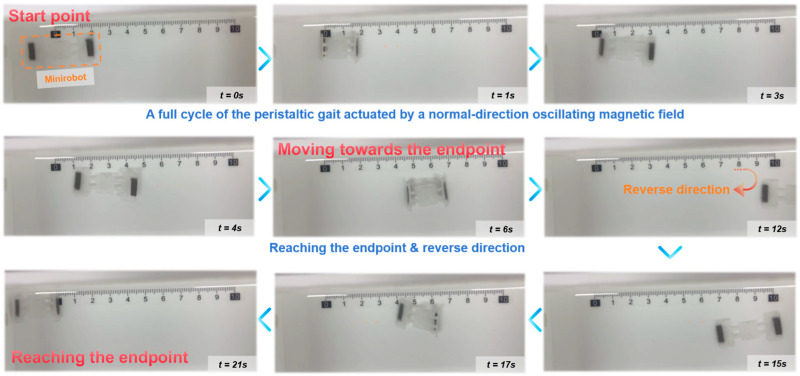
Time-lapse visualization of the peristaltic gait. The image sequence captures the robot executing a forward extension and contraction phase motion cycle on a planar surface driven by a vertically oscillating magnetic field. The robot moves from the start point, executing a directional reversal at the endpoint by inverting the magnetic signal phase, and successfully returning to the origin region.

Based on a quantitative analysis of the Video S1 footage, the biomimetic soft robot demonstrated stable bidirectional locomotion capabilities. The experimental result is shown in Figure 7 and Video S1. The motion trial commenced at t = 0 s, with the robot in a contracted resting state (Head: 2 cm; Tail: −1.5 cm). Driven by the external magnetic field, the robot executed a forward trajectory, reaching its turning point at t = 12 s. By calculating the displacement of approximately 2.0 cm relative to the tail’s initial position over this 5 s interval, the forward average velocity was determined to be approximately 0.9 cm/s. Following the turnaround maneuver at t = (12–14) s, the robot reversed its course and successfully returned to the origin region at t = 21 s, thus completing a closed-loop trajectory.

Mechanistically, the robot exhibits a clear flatworm or looper-like gait, relying on a stick-slip friction interaction with the substrate. The efficiency of this gait was particularly evident during the whole process, where a single extension-contraction cycle resulted in a significant forward stride. Data extraction from this interval indicates a stride length ranging between 0.8 cm, suggesting a high deformation-to-displacement ratio. In conclusion, the robot successfully validated its design by maintaining a linear path with a consistent velocity of 0.9 cm/s and demonstrating the capability for precise directional reversal within a 19 s round trip, highlighting its potential for navigation in confined environments.

### 3.6. Obstacle Crossing Experiment

**Figure 8:**
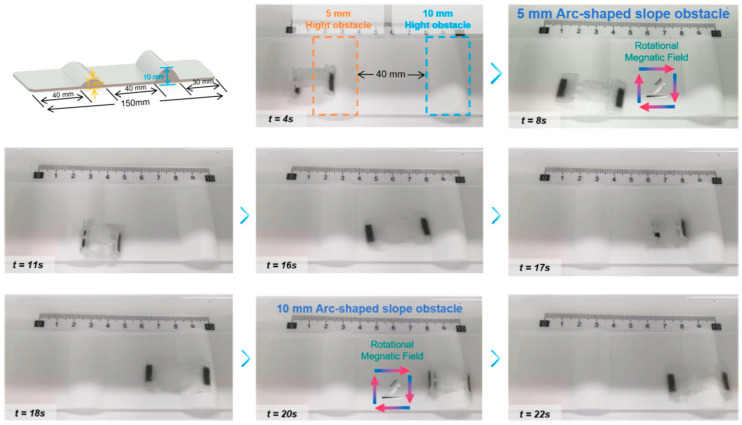
Demonstration of terrain adaptability and obstacle crossing. The robot traverses a 5 mm arc-shaped slope. The robot autonomously navigates a steep 10 mm obstacle (approx. 16 times its body thickness) using a high-lift strategy induced by a rotating magnetic field vector.

This experiment evaluated the terrain adaptability of the biomimetic soft robot by subjecting it to a round-trip test across obstacles of varying heights. The experimental setup featured two curved gradients with heights of 5 mm and 10 mm, respectively, separated by a 40 mm flat interval. As illustrated in Figure 8 and Video S2, the robot navigated this complex topography, maintaining a steady average velocity of approximately 0.5 cm/s on the flat sections. A key observation was the robot’s adaptive kinematic response when encountering the steeper 10 mm obstacle. In this phase, the robot autonomously adjusted its gait from a rapid forward inching motion to a high-amplitude lifting strategy, allowing the anterior magnetic segment to anchor onto the crest, thereby generating sufficient traction to overcome gravitational potential energy.

The successful locomotion over these obstacles can be attributed to the passive compliance of the robot’s soft body and the efficacy of the magnetic actuation. As the robot traverses the curved surfaces, its elastomeric body deforms to match the contour of the terrain, establishing conformal contact that maximizes friction and prevents slippage during the critical climbing phase. The ability to surmount an obstacle twice the height of the initial 5 mm barrier, followed by a controlled directional reversal and return journey, underscores the robustness of the system. These results demonstrate that the proposed robot possesses the mobility and stability required for inspection and navigation tasks in unstructured, confined environments such as biological lumens or industrial pipelines.

### 3.7. Load-Carrying Capacity Experiment

**Figure 9:**
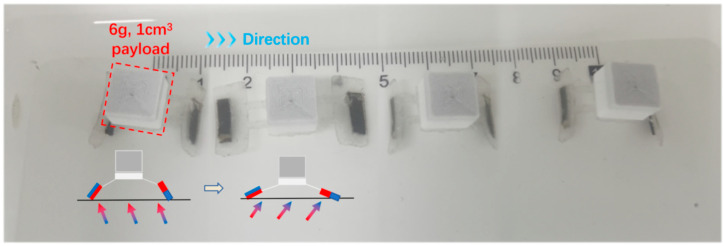
Experimental evaluation of load-carrying capacity. The image sequence demonstrates the robot transporting a 6 g cubic payload attached to its central flexible segment along a linear scale. Despite the significant increase in normal force and friction, the robot maintained a stable peristaltic gait, achieving an average forward velocity of approximately 0.69 cm/s. This performance, comparable to the unloaded velocity, confirms the high magnetic torque margin and the potential for targeted cargo delivery applications.

To demonstrate the practical cargo transport capability essential for applications such as drug delivery, a load test was conducted where a 6 g cubic payload was secured to the central bridge of the robot. The performance evaluation is shown in Figure 9 and Video S3. Under the same magnetic actuation frequency and intensity as the prior unloaded trials, the robot successfully navigated the track, covering a linear distance of approximately 9.0 cm in 13 s, corresponding to an average velocity of 6.9 mm/s. Notably, compared to the unloaded control group velocities of 4–7 mm/s, the addition of the 6 g payload did not result in any observable velocity degradation. This performance implies that the magnetic actuation provides a torque margin robust enough to overcome the increased inertia; furthermore, the additional normal force exerted by the payload likely enhanced the frictional anchoring of the robot’s feet during the stance phase, effectively reducing slippage. In addition, while the robot demonstrates sufficient magnetic torque to propel loads heavier than 6 g, the practical carrying capacity is primarily limited by the payload volume and center of mass rather than the actuation torque. Since metallic payloads would interfere with the driving magnetic field, non-metallic alternatives were required; however, at loads exceeding 7 g, the increased volume of these materials significantly raised the center of gravity, causing locomotion instability and frequent tipping. Thus, 6 g represents the maximum validated payload for stable, reliable navigation.

To evaluate the performance of the proposed robot within the current landscape of soft robotics, we conducted a benchmarking analysis against state-of-the-art crawlers, as summarized in Table 2. While comparable systems excel in specific metrics, such as the load capacity of multi-legged walkers, our flatworm-inspired design demonstrates exceptional agility and environmental compliance. It attains a maximum linear velocity of 11.1 mm/s, effectively outperforming comparable crawling prototypes. Critically, the robot demonstrates unprecedented terrain adaptability for a sheet-like crawler: the ability to traverse vertical obstacles 16 times its structural thickness represents a substantial improvement over traditional magnetic films.

## 4. Conclusions

This study presents the design, modeling, and characterization of a flatworm-inspired soft robot actuated by a staggered magnetization strategy. The proposed “active–passive–active” configuration effectively converts specific magnetic gradients into undulatory propulsion. A systematic investigation into the robot’s kinematic behaviors reveals that both static bending deformation and dynamic step length exhibit a linear correlation with the applied magnetic field strength. This linearity provides a predictable basis for locomotion control within the stable operational range.

Furthermore, the velocity analysis identifies a non-monotonic response to actuation frequency, with an optimal operating point observed at 7 Hz. This peak performance is attributed to the dynamic coupling between the structural resonance of the soft body and the driving frequency. Beyond this threshold, locomotion efficiency is attenuated by the material’s viscoelastic damping and phase lag in the passive segments. The consistency observed between the experimental data and the theoretical predictions validates the efficacy of the staggered magnetization mechanism and the hydrodynamic propulsion model.

In terms of adaptability, the robot demonstrates a 60 times payload-to-weight ratio, and the capability to traverse curved obstacles up to 16 times its body thickness. These metrics suggest significant advantages over traditional rigid robots in confined, unstructured environments. However, challenges remain for practical biological deployment. The current locomotion relies fundamentally on stick-slip friction interactions; consequently, performance may degrade in low-friction environments, such as mucus-covered tissues. Additionally, the current open-loop control system limits stability against high-flow fluid currents or external disturbances.

Future research will focus on addressing these limitations by integrating real-time magnetic localization to establish closed-loop control, thereby compensating for environmental uncertainties. Ultimately, this work provides a foundational framework for soft robotic interventions, with potential applications ranging from biomedical capsule endoscopy to industrial pipeline inspection.

## Figures and Tables

**Table 1:** Parameters of the Electromagnetic Manipulation Platform.

Parameters of the Electromagnetic Manipulation Platform	
Property	Parameter
Side length (mm)	200
Turns per coil	200
Magnetic field intensity (mT)	20
Copper wire diameter (mm)	0.8
Direct-current resistance (Ω)	10.7
Maximum current (A)	10.0
Magnetic field uniformity	≈9%
Center error	<1%
Magnetic field uniform region (Working space)	100 × 100 × 100 mm^3^

**Table 2:** Comparison of locomotion performance with related magnetic soft robots.

Reference	Robot Structure and Gait	Mass/Dimension	Max Velocity	Load Capacity (Rel. to Weight)	Obstacle Crossing Capability
Hu et al. [12]	Magnetic Film Sheet Undulation	~50 mg/20 mm	~4.0 mm/s	Low <10×	Low Step climbing
Kim et al. [21]	Ferromagnetic Domain Rolling/Crawling	Variable/~20 mm	Fast (Rolling) Slow (Crawling)	Low ~1–5×	Limited Mostly planar
Lu et al. [23]	Multi-legged Millipede Tapered Feet	~400 mg/15 mm	~3.0 mm/s Dry	Ultra-high ~100×	Good ~2–3× Body Height
Ze et al. [26]	Origami Crawler Earthworm Gait	~2 g/50 mm	~6.0 mm/s	Moderate ~10×	Moderate <1× Body Height
Ours	Soft Flatworm Traveling Wave	~328 mg/35 mm	11.1 mm/s	High ~20×	Excellent ~16× Thickness

## Data Availability

Data will be made available on request.

## Supplementary Materials

The following supporting information can be downloaded at https://www.mdpi.com/article/10.3390/biomimetics11010041/s1. The process of the experiment in this research can be found in Videos S1–S3.
